# A Comprehensive Review on Weight Gain following Discontinuation of Glucagon-Like Peptide-1 Receptor Agonists for Obesity

**DOI:** 10.1155/2024/8056440

**Published:** 2024-05-10

**Authors:** Ibrahim Abdullah bin Ahmed

**Affiliations:** Department of Family Medicine, Faculty of Medicine, Imam Mohammad Ibn Saud Islamic University, Riyadh, Saudi Arabia

## Abstract

Obesity is considered the leading public health problem in the medical sector. The phenotype includes overweight conditions that lead to several other comorbidities that drastically decrease health. Glucagon-like receptor agonists (GLP-1RAs) initially designed for treating type 2 diabetes mellitus (T2DM) had demonstrated weight loss benefits in several clinical trials*. In vivo* studies showed that GLP-1RA encourages reduced food consumption and consequent weight reduction by stimulating brown fat and enhancing energy outlay through the action of the sympathetic nervous system (SNS) pathways. Additionally, GLP-1RAs were found to regulate food intake through stimulation of sensory neurons in the vagus, interaction with the hypothalamus and hindbrain, and through inflammation and intestinal microbiota. However, the main concern with the use of GLP-1RA treatment was weight gain after withdrawal or discontinuation. We could identify three different ways that could lead to weight gain. Potential factors might include temporary hormonal adjustment in response to weight reduction, the central nervous system's (CNS) incompetence in regulating weight augmentation owing to the lack of GLP-1RA, and *β*-cell malfunction due to sustained exposure to GLP-1RA. Here, we also review the data from clinical studies that reported withdrawal symptoms. Although the use of GLP-1RA could be beneficial in multiple ways, withdrawal after years has the symptoms reversed. Clinical studies should emphasize the downside of these views we highlighted, and mechanistic studies must be carried out for a better outcome with GLP-1RA from the laboratory to the bedside.

## 1. Introduction

Around the world, obesity and its related comorbidities are one of the severe medical conditions concerning public health [1]. An overweight phenotype arises from obesity, which is a state of positive energy balance. This condition occurs when the energy consumed surpasses the energy used, leading to an accumulation of surplus calories in adipose tissue [2]. Related comorbidities include cardiovascular diseases, dyslipidemia, hypertension, type 2 diabetes, several forms of cancer, and many more [3–5]. Even though obesity leads to an intensified probability of illness and impairment, negatively impacting life expectancy and quality, weight reduction is correlated with a moderate enhancement in cardiometabolic indicators [6]. These include better control of blood glucose levels, elevation in high-density lipoprotein cholesterol and triglycerides, and improved blood pressure management among others [7, 8].

Healthy lifestyle and behavioral intrusions, calorie intake restrictions, increased energy expenditure, and weight loss surgery strategies could help reduce weight. However, discontinuing these lifestyle behaviors due to social, behavioral, financial, etc., make people unable to adhere to and complications after surgery results in weight gain [6, 9–11]. Beyond these methodologies, there has been substantial advancement in the sphere of pharmacotherapy for weight reduction. Yet, numerous antiobesity medications have noticeably fallen short due to their mediocre therapeutic impact and inferior performance over prolonged usage, coupled with intolerable side effects [12].

Scientists have identified various mechanisms that are crucial in controlling energy equilibrium and food intake patterns. These encompass the leptin-melanocortin trajectory, the opioid schema, the system involving glucagon-like peptide-1 (GLP-1) and its receptor (GLP-1R), and the axis of fibroblast growth factor 21 (FGF21) along with its receptor complex FGFR1c/*β*-klotho [13]. GLP-1 analogs and GLP-1R agonists are found to exert a reduction in weight and a hypoglycemic effect. As GLP-1 is readily degraded by dipeptidyl peptidase 4 (DPP-4) *in vivo*, several synthetic GLP-1 analogs were designed that are resistant to degradation, mimic the naturally occurring hormone GLP-1, and exert a stimulation effect on GLP-1R that are termed GLP-1R agonists (GLP-1RA). GLP-1RA stimulates insulin secretion and inhibits glucagon release. They have emerged as crucial elements in weight reduction by diminishing hunger pangs, mitigating food desires, decreasing caloric consumption, enhancing feelings of fullness, and promoting better control over eating habits [14, 15].

The effectiveness of medications observed in clinical trials does not show the same results in the real world, at least due to the lower adherence of patients, discontinuation of the therapy, and the lack of representativeness in participating in clinical trials. In addition, the other important factors that lead to cessation of discontinuation of GLP1-RAs or even unawareness of such therapies include inadequate health insurance coverage, restricted access to incretin-based treatments, budgetary limitations, insufficient health literacy, and obstacles between doctors and patients, such as provider bias [16].

Although randomized trials of GLP-1RA have demonstrated a meaningful outcome in weight change and other physiological aspects, GLP-1RAs are known to cause gastrointestinal disturbances, and they have been shown to negatively impact the adherence and persistence of their action when discontinued. Specifically, the question arises “Will there be a sustained weight loss after discontinuation of GLP-1 agonists for obesity treatment? [17].” In this review, we highlight the gaps found in the discontinuation of GLP-1RA and delineate the mechanism involved in the reversal of weight gain and other physiological targets involved in the development of obesity after discontinuation of GLP-1RA.

## 2. Overview of GLP-1R Agonists and Their Mechanism of Action in Weight Reduction

**Figure 1:**
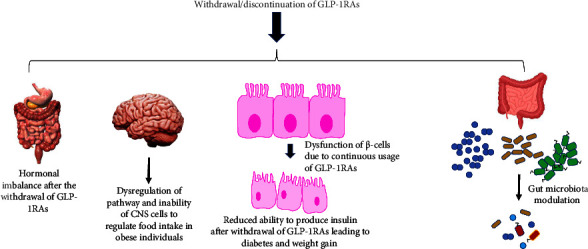
Mechanisms involved in weight gain post-withdrawal of GLP-1RAs.

The hormone GLP-1, a product of protoglucagon processing and produced by the endocrine cells in the intestinal epithelium, is an amino acid sequence comprising 30 to 31 units. It exerts its influence by promoting insulin secretion while concurrently reducing glucagon levels [2]. The effects of GLP-1 are transmitted via a solitary GLP-1R, which belongs to the G-protein coupled glucagon receptor family. Primarily, GLP-1Rs were identified within islet *β*-cells and the central nervous system (CNS) but later also located in various other regions including the stomach, small intestine mucosa, heart muscle tissue, and within enteric nervous system structures like Brunner's glands and blood vessels along with the sinoatrial node [17–20]. Through rigorous research on how GLP-1 interacts with its corresponding receptor—GLP-R—our understanding of their operational mechanism has been significantly expanded since they were first introduced for managing Type II diabetes (Figure 1). Nowadays however our knowledge has greatly extended beyond this original application due to ongoing research findings. Current applications see these receptor agonists being used more regularly for obesity management leading to decreasing appetite sensations and feelings of hunger while conversely increasing satiety post-consumption. They also affect gastric motility by slowing down food release from the stomach into the intestines [21].

### 2.1. GLP-RAs Control Body Weight/Energy Intake by Stimulating Different Regions and Pathways of the Brain Pathways

The first GLP-RA liraglutide was approved for treating obesity, as it promotes reduced food intake and weight loss. Research conducted on rodents by Nogueiras et al. showed that GLP-1RA activates brown fat and increases energy expenditure mediated through sympathetic nervous system (SNS) pathways [22]. Direct intracerebroventricular injection of GLP-1 into the mouse brain and recording sympathetic nerve activity in genetically manipulated mice lacking *β*-adrenergic receptors. This work showed CNS control of adipocyte metabolism involving SNS regardless of nutrient intake. van Can et al. reported that humans who were administered GLP-1RA (liraglutide) lost weight and experienced a modest shift toward fat oxidation and decreased energy expenditure, indicating that human weight loss is primarily a result of a reduction in food intake caused by GLP-1R agonism [23].

Several optogenetic and chemogenetic studies have been conducted to determine if Gcg and proglucagon-derived peptides (PGDP) are important for regulating food intake and glycemia in the brainstem. As a result of stimulating Gcg + neurons in the hindbrain, Gaykema et al. observed a reduction in food intake, metabolic rate, and glucose production [24]. This continuous activation of Gcg + neurons did not affect lean mice, while a decreased food intake was observed in mice fed a high-fat diet. Several PGDPs including glicentin, oxyntomodulin, glucagon, GLP-1, or GLP-2 may influence Gcg neuronal initiation [20, 25]. In response to GCG hypothalamic paraventricular nucleus (PVN) stimulation via Gcg activation, the brain stem showed anorectic responses toward excitatory neuronal circuits of the PVN plus corticotropin-releasing hormone (CRH) [26]. This is because neurons expressing Glp-1 transmit projections toward the paraventricular nucleus within the hypothalamus (PVN), playing a crucial role in controlling food consumption. In scenarios where glutamate is lacking, Liu and colleagues demonstrated how provoking Glp-1 sensory fibers located within the PVN can sufficiently decrease food intake without requiring glutamate discharge [26]. The report further revealed that activating the GLP-1 receptor (GLP-1R) intensifies excitatory synaptic efficacy in CRH neurons found in the PVN. The stimuli of GLP-1R set off a protein kinase A- (PKA-) dependent signaling sequence resulting in the phosphorylation of serine S845 present on GluA1 AMPA receptors followed by its transportation to the cell membrane. The findings suggest that a reduction of GLP-1R in PVN amplifies food consumption, leading to obesity. This outlines the critical function of GLP-1RAs in modulating dietary intake and managing weight gain.

### 2.2. GLP-1RA Regulates Food Intake through Stimulation of the Vagus Nerve GLP-1R

The vagus nerve has been identified as a vital conduit between our physiological systems and the brain, overseeing digestive, cardiovascular, and respiratory functions. It houses sensory neurons within the gut which perceive hormonal fluctuations and organ expansion [27]. In an attempt to demonstrate stimulation of vagal nerves by GLP-1 induction, Iwasaki et al. administered a noncalorific sweetener known as rare sugar D-allulose (D-psicose) to healthy animals as well as corpulent diabetic ones [28]. This team discovered that ingestion of D-psicose triggered vagal afferent signaling which in turn curtailed food consumption and enhanced glucose tolerance in mice subjects. Notably, these effects were diminished by either severing the vagus nerve or obstructing GLP-1R pharmacologically; likewise with gene deactivation of GLP-1R signaling throughout the body or selectively within vagal afferents. These findings provide valuable insights into how GLP-1RAs might influence vagal afferent GLP-1R signal transmission.

Similarly, Kreiger and his colleagues conducted an extensive study to illustrate the integral role of GLP-1 receptors within vagal afferent neurons (VANs), termed GLP-1R. Their method employed lentiviral-mediated knockdown of these receptors in male Sprague Dawley rats that led to an increase in food consumption and hastened gastric emptying, resulting in heightened postprandial glycemia as well as insulin secretion [29]. The interaction between GLP-1 and its receptor is instrumental not only for controlling food intake but also for managing obesity—a fact further consolidated by an earlier study undertaken by Sisley et al. [30]. In their research, they demonstrated how selective deletion of Glp1r located within visceral nerves coupled with the administration of liraglutide increased body mass or escalated food consumption among organisms consuming standard or high-fat diets. These particular investigations collectively imply that neuropathic interactions with liraglutide via neuronal GlpIR have significant effects on body weight regulation along with generating anorexic effects. As the vagus activity is increased by stimuli of gastric distension, cholecystokinin (released from the digestion of fat and proteins), and nutrients that induce satiety, it was expected that vagotomy would inhibit the signals reaching the CNS and would result in excess food intake [31].

### 2.3. Metabolites of the Microbiota in GLP-1RA Inflammation and Gut Microbiome

The significance of GLP-1 in the mitigation of inflammation is underscored because enteroendocrine cells (EECs) discharge GLP-1 not only as a result of the nutrient influx but also due to stimuli like interleukin-6, microbial by-products, lipid amides, and proteins, lipopolysaccharides (LPSs), and gut injury from ischemia [20]. In addition, a multitude of research has demonstrated that gut microorganisms can control satiety and glucose equilibrium by prompting the secretion of GLP-1. Similarly, it was discovered that the intestinal microbiota contributes significantly to human health through its regulation of body constitution, weight management, and diabetes prevention [32–34]. Research has indicated a significant impact of gut microbiota on aspects such as lipid processing, feelings of fullness, and abnormal fat deposition [32]. These findings led to the hypothesis that an analog of GLP-1 could deter weight accumulation by regulating gut microbiota. This was further validated by research carried out by Wang and colleagues, wherein the GLP-1RA known as liraglutide showed potential in altering gut microflora, thereby promoting lean-related characteristics aligning with weight reduction in mice with high blood sugar levels [25]. Further evidence was provided when Zhao et al. demonstrated alterations in gut flora triggered by liraglutide among simply overweight and T2DM overweight rats [35]. This suggested that GLP-1RA could impede weight increase through adjustments to the intestinal microbial population, including changes to its richness and diversity.

## 3. Factors Influencing Weight Regain after Taking Out GLP-1RA Treatment

During weight loss, several biological changes compensate for and prevent the maintenance of long-term weight loss, and weight regain is commonly observed [36]. Although lifestyle changes aid in improving weight management and help maintain weight loss, pharmacotherapy is indicated as an adjunct strategy for weight loss management. However, long-term pharmacotherapy is required to maintain weight loss and cessation or withdrawal from therapy leads to weight regain even with continuing lifestyle intervention [4]. Such observations have been made in those who had started GLP-1RA therapy and discontinued it. A few factors were identified as responsible for weight regain after cessation of treatment, including the CNS, deterioration of the activity of the cells secreting hormones, and transient hormonal adaptation to weight loss (Table 1).

### 3.1. Transient Hormonal Adaptation to Weight Loss

The balance between energy consumption and expenditure plays a pivotal role in body weight regulation. The CNS primarily the hypothalamus orchestrates this accord by interpreting peripheral hormonal signals that originate from an interconnected network of the gastrointestinal tract, pancreas, and adipose tissue [37]. Caloric limitation triggers compensatory responses such as significant downturns in energy usage alongside altered levels of leptin and cholecystokinin hormones. Furthermore, there is an observed surge in ghrelin hormone levels as well as appetite. In unison, these components stimulate weight rebound [38–40]. It has been posited that one potential mechanism underlying the phenomenon of weight regain or plateauing could be attributed to a decrease in leptin concentrations following initial weight reduction [38, 41]. Notably, it was discovered that sustaining free leptin quantities has implications for GLP-1RA-facilitated preservation of successful weight loss [41]. Yet it remains uncertain whether changes manifested during periods of reduced body mass involving hormones responsible for dictating appetite persist when maintaining lowered body mass over extended timeframes [38].

There has been a noticeable trend of quick weight regain following the discontinuation of semaglutide and other pharmacotherapies, as detailed in various trials [42]. The reason behind these fluctuations remains unclear; it is yet uncertain whether they denote a short-term compensatory reaction to an energy shortage. Interestingly, many studies have highlighted that several modifications prevail for up to a year post-weight loss [38] or even 6 years [43]. These investigations suggest the potential role of continued GLP-1RA administration in governing physiological and hormonal balances within the body. This regulation could be temporary and cease once treatment is halted, thereby leading to weight recovery after withdrawal.

### 3.2. Inability of CNS Cells to Regulate Weight Gain in the Absence of GLP-1RAs

The precise mechanism attributed to the link between GLP-1R signaling and body weight regulation in various species is difficult to understand due to multiple pathways regulating food intake and energy expenditure [44]. Intracerebroventricular injections and studies involving central administration of GLP-1 demonstrate the direct participation of the CNS in controlling food consumption and satiety, thus regulating body weight [20, 45]. The function of GLP-1 in the management of energy equilibrium is well established [46]. This peptide hormone is synthesized predominantly by L cells in the intestine and neurons residing in the nucleus of the solitary tract (NTS), located within the hindbrain [47]. Additionally, it has been suggested that other CNS cells, including microglia, may also contribute to GLP-1 production within the CNS milieu [48]. Parker and coworkers have shown that the levels of GLP-1 in plasma and the neurons that produce it increase in response to meal intake [49].

The central importance of the CNS in body weight regulation is substantiated by research conducted by Sisley et al. [30]. They employed Cre-Lox technology to selectively eliminate the GLP-1R gene within the CNS (nestin-Cre Glp1r^fl/fl^ mice) or peripheral nervous system (Phox2b-Cre Glp1r^fl/fl^ mice). Their findings indicated that for a comprehensive anorectic response to liraglutide, a long-lasting GLP-1R agonist, and for appreciating weight loss and anorectic effects associated with ongoing treatment using this agent, it is not vegal nerves but GLP-1Rs of the peripheral nervous system that are indispensable. Secher et al.'s work reinforces this viewpoint [50]. They adopted multiple methodologies such as injecting region-specific GLP-1 antagonists and conducting *ex vivo* electrophysiological examinations, along with administering peripherally a fluorescently tagged liraglutide molecule in rats. The outcomes demonstrated that proopiomelanocortin/cocaine and amphetamine-regulated transcript neurons (POMC/CART) are immediate targets of GLP-1RAs involved in weight reduction processes [50]. As the information provided by these studies shows the pathways involved, the withdrawal of GLP-1RA could have a deregulation effect on the CNS in controlling food intake and thus reversing weight gain [50]. Several studies report that the use of GLP-1RA for two years can have a profound impact on preventing weight gain [4, 51]. However, it is still necessary to confirm what adverse effects the continuous use of GLP-1RA would have on CNS cells and withdrawal. Will cessation of GLP-1RA after 2 years permanently impact regulating food intake and reduced body weight?

## 4. Limitations of the Review

This review has a few limitations. First, the data available regarding weight gain in patients after discontinuing GLP-1RA treatment are generally limited. Second, the mechanisms provided involved in weight gain in patients after withdrawal of GLP-1RA are only our point of view based on observations made by a few studies, and more conformational studies are required. Finally, we could provide only three different plausible mechanisms involved in the regain of weight after the discontinuation of GLP-1RAs, and there could be many that need to be further interrogated.

## 5. Conclusion

In the current situation, obesity and its related comorbidities are one of the severe medical obstacles. Many approaches have been strategized with the benefits of attaining weight loss, including diet, lifestyle and behavior change, and energy intake and expenditure restrictions. In addition to these, drug therapy, such as the use of GLP-1RA in medications for weight loss, has made significant progress. Obesity should be viewed not merely as an aesthetic issue, but as a persistent health condition. This perspective highlights the considerable contribution of medication in managing it. Capitalizing on over fifteen years of GLP-1 therapeutic applications, implementing these medications in addressing diabetes and obesity is progressively expanding. However, concerns are being raised regarding withdrawal symptoms from weight regain, comorbidities back to the baseline, and adverse effects due to prolonged use of these agonists. Studies delineating the mechanisms of the mode of action of GLP-1RAs in exerting weight loss and other ailments are commendable. Hence, it is imperative to conduct investigations on enhanced GLP-1 treatments with an accurate comprehension of effort mechanisms and a deeper insight into the withdrawal impacts and the conduct of cells that express GLP-1 receptors. With a similar concern in parallel, clinical studies are required to study the long-term safety of existing and new GLP-1RAs.

## Figures and Tables

**Table 1:** Clinical studies reporting withdrawal of GLP-1RA.

GLP-1RA used	Period of study	Weight loss observed (%)	Weight gain after withdrawal/continuity/placebo	Observations	References
Semaglutide	68 weeks	17.3	11.6	Withdrawal led to most of the weight loss with regain in 12 years. Cardiometabolic variables back to baseline requiring the need for continuous treatment	Lopez-Jimenez et al. [4]
Liraglutide	68 weeks (including 12 week off drug period)	6	1.9%	Liraglutide-induced weight loss combined with caloric restriction and lifestyle modification	Wadden et al. [52]
Semaglutide	20–68-week switch to placebo	7.9	6.9%	Maintaining treatment with semaglutide compared with switching to placebo resulted in continued weight loss over the following 48 weeks	Rubino et al. [53]
Liraglutide	54 weeks	5–10	At study end, weight had increased 0.53 kg with orlistat	In combination with diet, exercise, and behavioral modification, orlistat improved weight management in overweight adolescents compared with placebo	Le Roux et al. [54]

## Data Availability

All data used in the study will be available on request from the corresponding author.
